# Editorial: Genetic Regulatory Mechanisms Underlying Developmental Shifts in Plant Evolution

**DOI:** 10.3389/fpls.2019.00710

**Published:** 2019-06-04

**Authors:** Natalia Pabón-Mora, Verónica S. Di Stilio, Annette Becker

**Affiliations:** ^1^Instituto de Biología, Universidad de Antioquia, Medellín, Colombia; ^2^Department of Biology, University of Washington, Seattle, WA, United States; ^3^Institute of Botany, Justus-Liebig-Universität Gießen, Giessen, Germany

**Keywords:** plant development, evo devo, plant evolution, reproductive strategies, developmental modularity

The origin and evolution of land plants was accompanied by major macroevolutionary changes, including profound shifts in reproductive processes. Originally, plants were heavily dependent on water for gamete transfer, while later on, male gametes were dispersed by wind or animals within highly reduced gametophytes. Over time, female gametophyte development was internalized and sperm motility was lost, requiring a sperm delivery system reaching deep into the maternal tissues inside a megasporangium that evolved into the ovule in seed plants. Moreover, seed plant embryos became protected within the seed, which contains a nutritional start-up package for the next generation. In flowering plants, both gamete- and seed-dispersal mechanisms diversified. A carpel surrounded the ovules requiring fertilization to occur internally and endosperm developed with the embryo as a result of double fertilization, presumably reducing the risk of allocating resources to inviable seeds. Flowers developed unlimited displays for pollination, involving mostly variations in perianth presentation that include elaborate coloration and symmetry changes. Finally, as carpels mature into fruits, they showcase a wide array of forms that guarantee optimal seed dispersal.

Elaboration of the vegetative phase of land plants also played a major role in their evolution and habitat adaptation. For instance, fixed multicellularity in the diploid phase of the life cycle (i.e., embryo formation) marks the transition to land. Embryo patterning required the diversification of cell types, the polarization of stem cell niches in the diploid axis and the occurrence of lateral organs in response to auxin peaks to form leaf primordia. In the sporophyte, leaves develop from the Shoot Apical Meristem (SAM) achieving extreme morphological variation. Roots developing from a Root Apical Meristem (RAM) provided anchoring to the substrate and access to nutrients and water becoming another key innovation of the independent sporophyte. In parallel, the exposure to new environments triggered new symbiotic interactions.

Central genes that regulate each of these processes act together in larger gene regulatory modules and networks, and numerous components and interactions are known in the model plant *Arabidopsis thaliana* (thale cress). However, how the gene regulatory modules have arisen over evolutionary time to enable these evolutionary transitions remains an open question at the core of plant evolutionary developmental research studies. This special issue collects articles contributing to this question from different perspectives and systems.

## Origins of Complexity: Theoretical Approaches and the Study of Gene Evolution Over Deep Phylogenetic Timescales

Complexity in plant body patterning has been addressed from both morphological and genetic standpoints in this issue. The article by Benítez et al. aims to understand the mechanisms responsible for the major body plan transitions in green algae belonging to different clades and the monophyletic land plants. They introduce the concept of dynamical patterning modules (DPMs) which are defined as sets of conserved gene products and molecular networks, in conjunction with the physical morphogenetic and patterning processes where they function. They note that critical DPMs for the evolution of plants are cell-to-cell adhesion, placement of the cell wall, cell differentiation, and cell polarity. Analyzing data from broad phyletic comparisons, they conclude that the same DPM patterns have emerged many times independently, and that unicellular plant ancestors already possessed most molecular mechanisms later co-opted by multicellular plants, regardless of whether the diploid or haploid life phase is the dominant one. They further show that plasmodesmata were critical for the evolution of complex multicellularity in plants.

Yruela et al. highlight the evolution of protein ductility (intrinsic disorder) in unicellular and multicellular organisms. Investigating the occurrence of protein ductility associated with gene duplication led them to conclude that it increases in concert with organismic complexity. Moreover, the distribution of intrinsically disordered proteins and residues is not random but can be correlated with chromosomal rearrangement during evolution.

Complexity can also be studied in terms of gene regulation over developmental or evolutionary timescales. Bräutigam and Cronk examined the role of DNA methylation in gene regulation to fine-tune developmental plasticity. DNA methylation plays a role in enlarging the potential of phenotypic expression and the authors argue for the emergence of the novel research field of “epi-evo-devo.” Complexity can also be modulated by changes in developmental timing; Buendia-Monreal and Gillmor set up a framework in which alterations in the timing of developmental programs during evolution (also known as heterochrony) led to changes in the diversification of key innovations such as leaves, roots, flowers and fruits.

Synapomorphies in plant evolution can also be studied from the evolutionary perspective of gene lineages controlling developmental, morphological and physiological changes. For example, *AINTEGUMENTA* genes encoding *AP2*-type transcription factors play multiple functions in plant development including the maintenance of stem cell niches, embryo patterning, lateral organ formation, and fatty acid metabolism. Dipp-Álvarez and Cruz-Ramírez provide a comprehensive evolutionary framework for the *ANT* gene lineage across streptophytes, where *preANT-like* genes are present in the ancestor of embryophytes and a gene duplication occurs in land plants resulting in the *basalANT* and the *euANT* lineages. Possible roles facilitated by these transcription factors during the transition to dry environments include enhanced tolerance to desiccation, and the establishment and maintenance of multicellularity.

The above-mentioned articles contribute to a theoretical and experimental foundation for assessing how major novelties may have arisen during plant evolution, and how potential fine-tuning of the genetic components responsible for a trait can lead to considerable variation in plant body plans.

## Origin and Patterning of the Gametophyte and Sporophyte in Early-Diverging Land Plants

Three manuscripts focused on early-diverging lineages, including non-vascular bryophytes and vascular lycophytes. Flores-Sandoval et al. provide an overview of transcription factors controlling developmental transitions during the life cycle of the model liverwort *Marchantia polymorpha*. The authors explore differentially expressed genes during specific developmental time points, resulting in sets of genes acting exclusively in either gametophyte or sporophyte, in the reproductive transition or in antheridia or archegonia development. Their analyses reveals auxin co-expression groups present in liverworts and mosses that are not dependent on the single class-C Auxin Response Factor (ARF), which in other plant groups is directly involved in auxin responses. Moreover, their data confirm the participation of *MpARF3* as a negative regulator of reproductive transition.

The article by Grosche et al. shows that the common symbiotic pathway required for the mutualistic interactions between plants and mycorrhiza and the downstream GRAS-domain transcription factors important for its establishment were already present in the land plant ancestor. Using phylogenetic reconstructions, they showed moss lineage specific gene losses and expansions as well as absence of symbiotic GRAS TFs in algae. The ancestral presence of symbiotic GRAS TFs may have therefore provided a platform for conquering the land by enhancing microbial interactions. This idea is supported by the fact that in mosses, gene losses in some lineages coincide with lack of arbuscular mycorrhiza symbiosis.

A different approach was taken by Augstein and Carlsbecker who were able to trace roots into two independent origins in lycophytes and euphyllophytes (which include ferns and seed plants). They review the anatomical diversity of roots, emphasizing stele patterning, the auxin pathways in the RAM in different taxa, the genetic mechanisms involved in stem cell niche maintenance and the root cap control. Most data gathered so far comes from Arabidopsis, other Brassicales, the ferns *Azolla filiculoides* and *Ceratopteris richardii* and the lycophyte *Selaginella moellendorfii*. However, the authors emphasize that functional tools for comparative analyses are needed in lycophytes and ferns in order to establish the conservatism of such networks across vascular plants.

## Evolution of the Megasporangium (ovule) and Megagametophyte in Seed Plants

Gymnosperms showcase unique reproductive features that include the nourishment of the embryo directly by the female gametophyte and the nucellus, which play the equivalent role of the endosperm in angiosperms. Moyano et al. describe the dismantling of the megagametophyte during germination of *Araucaria angustifolia* seeds, focusing on the mechanisms activating programmed cell death (PCD) and the availability of proteins, starch and lipid bodies for the developing embryo.

Ovule integuments are another distinctive feature distinguishing gymnosperms, which have one, from angiosperms, which have typically two. Integuments protect the ovules and seeds, define a route for pollen entry and contribute to seed hydration and dormancy. In an effort to assess the molecular basis of integument identity Arnault et al. investigate the expression of integument genes in the early-diverging angiosperm *Amborella trichopoda* and conclude that *YABBY, KANADI*, and *HD-ZIPIII* transcription factors have conserved expression patterns between *Amborella* and *Arabidopsis*. Their data contribute to the reconstruction of molecular mechanisms for integument identity during the evolution of angiosperms.

Akhter et al. explore the evolution of gene lineages that exclusively expanded in gymnosperms compared to angiosperms employing RNA sequencing. They focus on the *TM3-like* MADS box genes, from which *DAL19* has been previously identified as being specifically upregulated in cone-setting shoots. They show the importance of previously unrecognized, and sometimes mutually exclusive, mRNA splice variants in *Picea abies*. They also highlight isoforms that are differentially expressed in male and female cone meristems, as well as in vegetative meristems. From their work, we derive that splice variants are in fact another source of variation contributing to functional evolution, working in parallel with gene duplication.

## Evolution of Molecular Mechanisms Underlying Flowering

Gene duplication is a known source for variation and functional diversification, triggering major evolutionary shifts. Flowering is one of the most important transitions leading to angiosperms; during this process, the SAM becomes an inflorescence meristem that in turn develops floral meristems in its flanks. Lee et al. exemplify the role of gene duplication in one of the key players in the transition to flowering, *FLOWERING LOCUS C* (*FLC*) in *Boechera stricta* (Brassicaceae), resulting in the acquisition of unique roles in the different paralogs. While one of the paralogs plays a conserved role delaying flowering, the other has lost its flowering function altogether. The authors uncover independent mutations that change the species phenology and provide evidence for heritable variations in vernalization requirements and flowering time via FLC in Brassicales.

A mini review by Monniaux and Vandenbussche discusses perianth evolution. They propose that the perianth is formed in the outer floral whorls to maintain the stamen and carpel identity gene's expression exclusively in the center of the flower. They evaluate negative regulators of B and/or C-class genes, especially of the *APETALA2* (*AP2*) type and highlight the need for a broader comparative framework including early-diverging angiosperms with and without perianth. Exploring the molecular mechanism underlying floral organ identity Galimba et al. investigate the genetic redeployment of B-class genes in apetalous *Thalictrum* (Ranunculaceae). Ranunculaceae petals have been lost repeatedly, presumably in conjunction with the loss of the petal-specific *AP3-III* paralog. The authors present evidence for partial redundancy for stamen identity in the remaining paralogs and a role for these genes in the ectopic petaloidy of sepals, while proposing a novel mechanisms of dominant-negative regulation of B-class genes by a truncated *AP3-II* ortholog.

Damerval et al. explore the genetic underpinnings of floral symmetry changes affecting floral display in Proteaceae, an early diverging eudicot family. They find that in *Grevillea juniperina*, adnation of stamens to tepals and asymmetrical growth of the single carpel contribute to the establishment of floral bilateral symmetry. An annotated floral transcriptome for *G. juniperina* is also presented, with an emphasis on floral MADS-box genes and TCP Class I and Class II gene expression patterns, the latter known to control floral bilateral symmetry in core eudicots.

Contributing to a more conceptual understanding of floral symmetry control, Sengupta and Hileman discuss the idea of direct transcriptional autoregulation (DTA) of *CYCLOIDEA* (*CYC*) genes. They present *in silico* predictions and experimental evidence for DTA in flower symmetry evolution and propose that *CYC* autoregulation may have evolved via *de novo* mutations and could have played a key role in the origin of monosymmetric flowers in the Lamiales.

## Evolution of the Genetic Network Controlling Fruit Development

A fruit genetic network is well established in Arabidopsis, but comparative analyses of the key players controlling histogenesis during fruit development outside Brassicaceae is still scarce. Zumajo-Cardona et al. present the evolution of the *REPLUMLESS* (*RPL*) gene lineage, focusing on the expression patterns of *RPL* homologs in Papaveraceae (a basal eudicot). Arabidopsis *RPL* controls the identity of the replum, a medial persistent fruit layer unique to Brassicaceae fruits. In contrast, *RPL* homologs control fruit shedding in rice, whereas in poppy they are broadly expressed during flower development and become restricted to the dehiscence zone during fruit maturation, suggesting shifting roles of *RPL* genes during angiosperm diversification.

Maheepala et al. present a characterization of Solanaceae *FRUITFULL* (*FUL*) genes. *FUL* is responsible for fruit wall proliferation and for limiting the dehiscence zone in the Arabidopsis silique. While *FUL* homologs play the same roles in dry-fruited Solanaceae, they have taken on new roles in fleshy fruit development where they regulate aspects of the ripening processes, such as pigment accumulation. The authors show that Solanaceae have four *FUL* paralogs, some originating as a result of a whole genome multiplication event, others by tandem duplication and one clade even undergoing pseudogenization. While some Solanaceae *FUL* clades appear to have acquired novel functions in fleshy fruit development, the molecular mechanisms underlying the *FUL* function shifts require additional analyses.

In summary, this research topic explores the genetic mechanisms controlling key developmental transitions, both vegetative and reproductive, during plant evolution. It includes original contributions on a variety of scales and processes: from factors contributing to multicellularity, to body plan complexity, developmental plasticity, heterochrony, tolerance to desiccation, gametophyte to sporophyte transition, establishment of embryo polarity and elaboration of the apical and root meristems and symbiotic interactions in early diverging land plants. Recognizing that reproductive shifts have also occurred in more recent phylogenetic scales, this collection also includes manuscripts focusing on the control of flowering, the development of ovules and perianth, floral symmetry and display and the elaboration of fruits from carpels aimed at dispersing the next generation. We hope that such comprehensive overview will be inspiring and will motivate additional efforts in the scientific community to continue to explore these processes holistically across land plants.

## Author Contributions

NP-M wrote the first draft of this manuscript, VSD and AB revised it and completed the text. All authors made direct intellectual contribution to the work and approved it for publication.

### Conflict of Interest Statement

The authors declare that the research was conducted in the absence of any commercial or financial relationships that could be construed as a potential conflict of interest.

